# Human iPSC‐Derived Microglia Integrate Into Cerebral Organoids and Assume an In Vivo‐Like Phenotype

**DOI:** 10.1111/ejn.70281

**Published:** 2025-11-12

**Authors:** Emile Wogram, Felix Sümpelmann, Andrew Khalil, Anthony Flamier, Dongdong Fu, George W. Bell, Rudolf Jaenisch

**Affiliations:** ^1^ Whitehead Institute for Biomedical Research Cambridge Massachusetts USA; ^2^ Institute of Neuropathology, Faculty of Medicine University of Freiburg Freiburg Germany; ^3^ The Wyss Institute for Biologically Inspired Engineering Boston Massachusetts USA; ^4^ Harvard John A. Paulson School of Engineering and Applied Sciences Boston Massachusetts USA; ^5^ Department of Biology Massachusetts Institute of Technology Cambridge Massachusetts USA

**Keywords:** cerebral organoids, human stem cells, microglia, RNA‐seq

## Abstract

Microglia, the brain‐resident macrophages, are critically involved in numerous physiological and pathological brain processes, including neoplasms, epilepsy, and neurodegeneration. However, investigating microglial function is notoriously difficult because they are extremely sensitive to changes in their environment and drastically alter their transcriptional state and morphology once they are isolated from the brain and cultured in vitro. In vivo experiments in mice are likewise limited because of vast differences between mouse and human microglia, particularly regarding the expression of disease‐associated genes. To overcome this issue, we developed a highly controlled in vitro cerebral organoid platform where human microglia adopt an in vivo‐like phenotype. This approach allows long‐term studies and high‐throughput analysis of in vivo‐like human microglia suitable for disease modeling and drug testing.

## Graphical abstract

Microglia, the resident macrophages of the brain, play important functions in health and disease, yet it is difficult to study human microglia in vitro. In this study, we developed a highly controlled in vitro cerebral organoid platform where human microglia adopt an in vivo‐like morphology and gene signature. This setup allows long‐term studies and high‐throughput analysis of in vivo‐like human microglia suitable for disease modelling and drug testing.

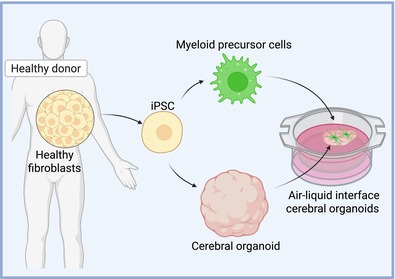

**Abbreviations**
3D: three‐dimensional
7d pM: primary human microglia cultured 7 days in vitro
ad
: Alzheimer's disease
ALI‐CO: air‐liquid interface cerebral organoids
DIV: days in vitro
EGFP: enhanced green fluorescent protein
ESCs: embryonic stem cells
FACS: fluorescence‐activated cell sorting
hPSCs: human pluripotent stem cells
iMG: hPSC‐derived microglia
iMPC: myeloid precursor cells
in vivo‐iMG 10d/ 60d: iMG transplanted into mice for 10 days/60 days
iPSC: induced pluripotent stem cells
LPS: lipopolysaccharide
PCA: principal component analysis
pMG: primary microglia freshly isolated from human patients' brains
pMono: primary monocytes

## 1 Introduction

Microglia are the immune‐competent cells of the brain and play crucial roles during brain development, brain homeostasis, and in pathological conditions, including brain cancers, neuroinflammatory diseases, and epilepsy (Wolf et al. 2017; Avignone et al. 2008; Kettenmann et al. 2011). Microglia sense minute changes in their environment upon which they rapidly and drastically alter their phenotype (Hanisch and Kettenmann 2007). This microglia characteristic introduces challenges for studying them in vitro, as primary microglia rapidly adopt a vastly different transcriptional profile within hours of being cultured outside patients' brains (Gosselin et al. 2017). Similarly, the phenotype and expression profile of cultured microglia derived from human embryonic stem (ES) or induced (iPSC) pluripotent stem cells (hPSCs) more resemble in vitro human microglia than homeostatic in vivo microglia directly isolated from the brain (Svoboda et al. 2019). Additionally, previous studies have demonstrated that cultured cells, but not ex vivo cells, aberrantly express a number of disease‐relevant microglia genes, such as Alzheimer's disease (AD) risk variants, which are less expressed in homeostatic microglia residing in the brain (Gosselin et al. 2017). Recent studies have shown that when placing human iPSC‐derived microglia into the in vivo brain environment using immunocompromised newborn mice, these cells integrate throughout the brain and assume a state very similar to the homeostatic state of microglia in the human brain (Svoboda et al. 2019; Hasselmann et al. 2019). These results indicated that hPSC‐derived microglia (iMG) can adopt the transcriptional state and cellular phenotype of microglia residing in the healthy brain. However, significant limitations of this interspecies chimera approach to studying microglia include variability of engraftment, low throughput, and that these human microglia interact with neurons and glia of a different species. To avoid these limitations, other studies have aimed at incorporating human iMG into cerebral organoids to create a fully human and isogenic three‐dimensional (3D) environment (Brownjohn et al. 2018; Bodnar et al. 2021; Fagerlund et al. 2021; Hong et al. 2023). Although these studies demonstrated the successful integration of microglia into cerebral organoids, microglia contribution to the organoids is typically rather sparse and inhomogeneous, lack the highly ramified morphology typical for homeostatic microglia in vivo, or transcriptional analyses comparing their cell identities to postnatal, primary human microglia are largely absent (Brownjohn et al. 2018; Park et al. 2023; Lin et al. 2018).

With the aim to better study human microglia biology for disease modelling and therapy development, we created for the first time a cerebral organoid culture system in which human microglia integrate evenly throughout the tissue and adopted an in vivo‐like phenotype within a few days. Our results of cerebral organoids containing myeloid‐derived microglia provide a potentially powerful tool for studying human microglia biology in health and disease. Specifically, the system described here allows long‐term studies of microglia biology with cells with different genetic backgrounds, live imaging, high‐throughput analysis, and pharmacological testing in a highly controlled fashion.

## 2 Results

### 2.1 Generation of Cerebral Organoids at the Air‐Liquid Interface Containing iMG

**FIGURE 1:**
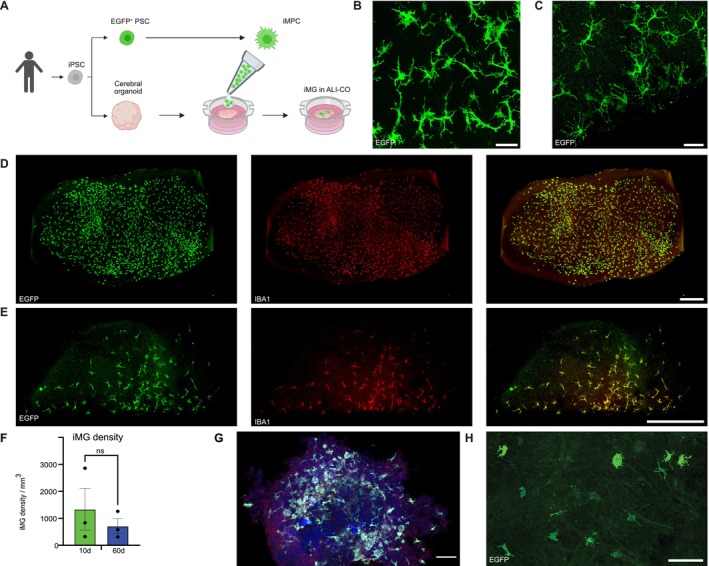
Human iMG migrate and disperse in ALI‐COs and adopt an in vivo*‐*like morphology. (A) Experimental schematic showing the reprogramming of foreskin fibroblasts of a healthy 2‐year old male to iPSCs and either differentiation to cerebral organoids or genetically integrating EGFP in their genomes for the generation of EGFP‐expressing microglia progenitor cells (iMPC). Cerebral organoids were sliced and placed in transwells after 100 days of differentiation. After 7 days of recovery, iMPC were added in suspension onto the ALI‐COs in a drop‐like fashion. Created with BioRender.com. (B, C) EGFP‐expressing iPSC‐derived microglia (iMG, green) with a highly ramified morphology residing in ALI‐COs for 10 days (10d) (B) and 60d (C). Scale bars: 50 μm. (D, E) iMG expressing EGFP (green) and Iba1 (red) in ALI‐COs (D: 10d, E: 60d). Scale bars: 0.5 mm. (F) Quantification of iMG density within ALI‐COs (*n* = 3, two‐tailed *t*‐test, plotted are the mean with standard error of the mean). (G) iMG added onto an uncut cerebral organoid and cultured for 2 weeks. EGFP (green, no primary antibody was added, as EGFP transgene expression was sufficient for image acquisition), MAP 2 (blue), TUJ1 (red). Scale bar: 100 μm. (H) ALI‐CO‐iMG 10d after a 48 h challenge with 100 ng/mL LPS. Scale bar: 100 μm.

To create an optimal in vitro environment supporting the development of in vivo‐like human iMG, we differentiated hiPSCs into cerebral organoids. To prevent the growing organoids from developing a hypoxia‐induced necrotic core, which would affect the microglia phenotype, we sectioned the organoids monthly, starting 60 days after organoid induction. After they matured for 100 days in vitro (DIV), we placed the slices on transwells, which supplied them with fluids and nutrients from the bottom and oxygen from the top (Figure 1A). This method was adapted from recently published work demonstrating that air‐liquid interface cerebral organoids (hereafter referred to as ALI‐COs) exhibit greatly improved cell survival and morphology compared with uncut organoids (Giandomenico et al. 2019). After a week of recovery, myeloid precursor cells (iMPC), derived from isogenic hiPSCs with enhanced green fluorescent protein (EGFP) inserted into the *AAVS1* locus, were added on top of the ALI‐COs using an established and characterized cell line (Wogram et al. 2024, Figure 1A).

Within a day, the cells began migrating into the tissue, where they evenly distributed and adopted a ramified morphology within 10 days, after which they were termed “iMGs,” as they developed a microglia‐like morphology (Figure 1A,B). Hence, ALI‐COs contained major brain cell types. In addition to iMG, the ALI‐COs contained SMI312‐expressing neurons (Figure S1A), Synapsin1 positive structures indicative of synapses (Figure S1B), nestin‐expressing neural precursor cells (Figure S1C), and MBP and Olig2 expressing oligodendrocytes (Figure S1D). After 60 days in ALI‐COs, iMG became further ramified (Figure 1C,E), while iMG density in ALI‐COs decreased insignificantly from 1339 ± 774 cells/mm^3^ to 714 ± 282 cells/mm^3^ (Figure 1F). This iMG density is about 15.6% to 8.4% of the microglia cell density in the human cortex, respectively, where the density is 8500 ± 3900 cells/mm^3^ (Keller et al. 2018); yet these numbers change during development and aging (Menassa et al. 2022).

Notably, iMPCs added to uncut cerebral organoids adopted an amoeboid morphology (Figure 1G). Next, we tested whether iMG reacted to an inflammatory stimulus by treating them with lipopolysaccharide (LPS). After 48 h of exposure, the cells' morphology changed drastically from a ramified morphology to an amoeboid‐like morphology with short, thick cell processes and enlarged somata (Figure 1H).

### 2.2 iMG Adopt a More Ramified Cell Morphology in ALI‐COs

**FIGURE 2:**
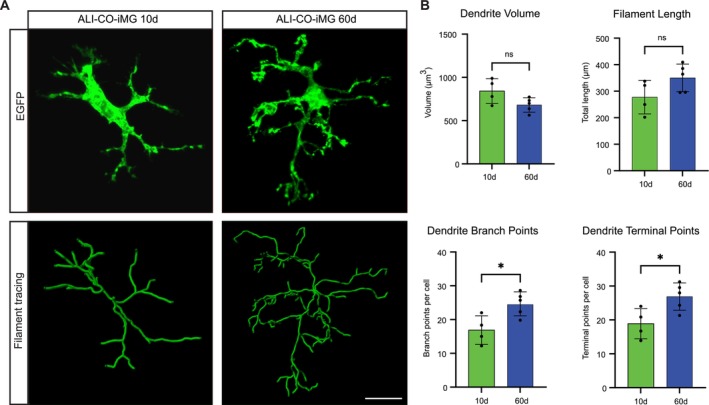
iMG adopt a more ramified cell morphology within ALI‐COs. (A) A representative iMG before (upper row, confocal microscopy, *Z*‐projection) and after (bottom images) filament tracing after 10 days (left images) and 60 days in ALI‐COs (right images). Scale bar: 20 μm. (B) Quantification of changes in ALI‐CO‐iMG 10d (10d, green bars) and 60d (60d, blue bars) iMG cell morphology by analyzing dendrite volume, filament length, dendrite terminal points, and dendrite branch points. Significance was determined with an unpaired *t*‐test, with sample sizes of 46 to 107 cells from three organoid sections. *p < 0.05; ns, not significant. Data are represented as mean ± standard deviation.

Beyond the qualitative assessment of ramification, we performed an in‐depth analysis of iMG morphology to detect morphological differences in iMG cultured for 10 or 60 days (ALI‐CO‐iMG 10d or 60d, respectively). Filament tracing of 46 to 107 cells from different ALI‐COs using high‐resolution images (Figure 2A) revealed changes in overall dendrite volume, filament length, dendrite terminal points, and branch points. The dendrite branch points and terminal points increased significantly over prolonged culture duration (Figure 2B). Notably, in ALI‐CO‐iMG 60d, dendrite volumes (681.5 ± 82.84 μm^3^), filament lengths (350.1 ± 52.14 μm), branch points (24.66 ± 3.54), and terminal points (26.89 ± 4.02) were in similar ranges as measured in human iMG 60 days after transplantation into neonatal mouse brains using the same analysis (Svoboda et al. 2019).

In summary, iMG became more ramified over time inside ALI‐COs. This is reminiscent of the maturation of microglia in vivo, which become more ramified as they mature (Cengiz et al. 2019; Nikodemova et al. 2015). Lastly, similar to homeostatic microglia in the brain, live imaging of ALI‐CO‐iMG 10d and 60d revealed that these iMG have multiple processes that constantly move, while their soma was stationary (Movies S1 and S2).

### 2.3 iMG Adopt an In Vivo‐Like Transcriptional State in ALI‐COs

To compare cell identities of iMG in ALI‐COs to primary human microglia, we analyzed the transcriptional profiles of iMG after 10d and 60d. EGFP‐expressing cells were isolated via *fluorescence‐activated cell sorting* (FACS) and subjected to bulk RNA sequencing. In addition, we performed RNA sequencing of iPSC‐derived myeloid precursor cells (iMPCs) and iMG cultured in vitro for 10 and 60 days (10 or 60d iMG). We compared gene expression profiles of these conditions with publicly available RNA sequencing data from primary microglia freshly isolated from human patients' brains (pMG) or primary human microglia cultured 7 DIV (7d pMG), and primary monocytes (pMono) (Gosselin et al. 2017). In addition, we included published transcriptional profiles in our analysis, from iMG transplanted into mice for 10 days (in vivo‐iMG 10d) and 60 days (in vivo‐iMG 60d) which represent iMG in an in vivo setting (Svoboda et al. 2019).

**FIGURE 3:**
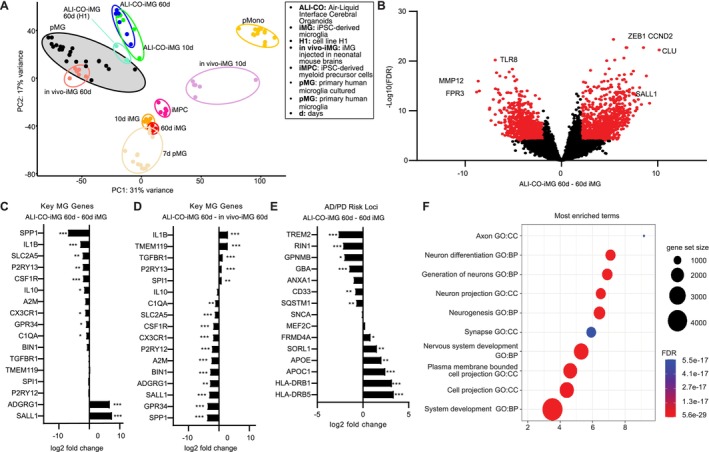
iMG adopt an in vivo‐like transcriptional state in ALI‐COs. (A) Principal component analysis (PCA) using top 2500 features of iMG and control cell types. ALI‐CO‐iMG 60d, iMG cultured in ALI‐CO for 60 days; ALI‐CO‐iMG 10d, iMG cultured in ALI‐CO for 10 days; ALI‐CO‐iMG 60d (H1), iMG from cell line H1 cultured in ALI‐CO for 60 days; in vivo‐iMG 60d, iMG 60 days after injection into neonatal mouse brains; in vivo‐iMG 10d, iMG 10 days after injection into neonatal mouse brains; iMPC, iPSC‐derived myeloid progenitor cells; 10d iMG, iMG cultured for 10 days in monoculture; 60d iMG, iMG cultured for 60 days in monoculture; pMono, primary human monocytes; pMG, primary human microglia; 7d pMG, primary human microglia cultured for 7 days in monoculture. Lines to distinguish clusters were drawn by hand. (B) Volcano plot of detected genes depicting the ratio of expression between ALI‐CO‐iMG 60d and 60d iMG. Gene expression is illustrated as the ratio of log2‐transformed normalized counts (x‐axis) and log10‐transformed normalized counts (*y*‐axis). Red dots are significantly differentially expressed genes (FDR < 0.0001, log2 fold change > 2). A few relevant microglia genes are annotated. Differential gene expression analysis of genes relevant to microglia development and homeostasis (C–D), and ad/PD risk genes are plotted in ALI‐CO‐iMG 60d vs. 60d iMG, or ALI‐CO‐iMG 60d vs. in vivo‐iMG 60d (E). *p < 0.05; **p < 0.01; ***p < 0.001; no significance is not specified. (F) Gene ontology enrichment analysis of numerous biological processes significantly upregulated in ALI‐CO‐iMG 60d over 50 DIV iMG.

Notably, principal component analysis (PCA) revealed that pMG showed higher similarity (*p* < 0.00001) to iMG in ALI‐COs (ALI‐CO‐iMG 10d and 60d) than to iMG cultured in vitro (10 and 60d iMG; Figure 3A). As expected, the transcriptional profiles of iMPC and pMono, as well as 7d pMG, were more different (*p* < 0.00001) compared with pMG. Notably, iMG adopted a more in vivo‐like gene signature within 10 days after transplantation into ALI‐COs (ALI‐CO‐iMG 10d) in contrast (*p* < 0.00001) to iMG transplanted into mice, which required a more extended period (in vivo‐iMG 60d, compared with in vivo‐iMG 10d) to acquire an in vivo‐like gene signature. This same change was observed in independent biological replicates and human ESC‐derived microglia added to isogenic ALI‐COs (ALI‐CO‐iMG 60d (H1); Figure 3A).

Direct comparison of individual differentially expressed genes revealed that several genes relevant to microglia biology were upregulated in ALI‐CO‐iMG 60d, compared with iMG 60d iMG (Figure 3B,C). This included genes like *ADGRG1*, a gene involved in cortical development, and *SALL1*, a key homeostatic microglia transcription factor defining microglia identity and function, which were highly and significantly upregulated in ALI‐CO‐iMG 60d and expressed at similar levels as in primary microglia (Buttgereit et al. 2016). In contrast, other genes, including *SPP1*, an interferon‐gamma and interleukin‐12 regulator, were strongly downregulated. Compared with iMG transplanted into mice for 60 days (in vivo‐iMG 60d), 60d microglia upregulated the homeostatic marker gene *TMEM119*, but also the proinflammatory gene IL1B and anti‐inflammatory gene *TGFBR1* (Figure 3D). On the other hand, in vivo‐iMG 60d showed higher expression values for homeostatic markers such as *P2RY12* or *CX3CR1*, as well as *SPP1*, which is upregulated by macrophages around vasculature, which was lacking in our culture system (Hong et al. 2023; De Schepper et al. 2023).

Genes linked to neurodegenerative diseases, such as *GPNMB*, *GBA*, *CD33*, and *TREM2*, were downregulated in ALI‐CO‐iMG 60d compared with 60d iMG. In contrast, other genes such as *APOC1* and *APOE* were upregulated in ALI‐CO‐iMG 60d (Figure 3E). Also, *HLA‐DRB5* and *HLA‐DRB1*, genes involved in the presentation of peptides derived from extracellular proteins, were upregulated in iMG in ALI‐COs. We performed gene ontology analysis to further elucidate functional differences between ALI‐CO‐iMG 60d and 60d iMG. Among the top GO Terms were “nervous system development,” “neurogenesis,” and “neuron differentiation,” significantly and strongly enriched in iMG in organoid ALI‐COs, implying that iMG do interact with their neuronal environment (Figure 3F). In summary, iMG in ALI‐COs upregulated key homeostatic microglia genes and downregulated several neurodegeneration‐associated genes compared with iMG in monoculture and more closely interacted with their microenvironment.

## 3 Discussion

Microglia shape the brain during development and homeostasis and play central roles in many brain pathologies (Huang et al. 2017; Nott et al. 2019; Novikova et al. 2021; Podlesny‐Drabiniok et al. 2020; Wightman et al. 2021). However, the difficulties in studying microglia in health and disease are manifold, as they react quickly to subtle experimental manipulations and show differences across species. Recently, our lab and others have succeeded in differentiating microglia from human PSCs in a highly efficient and robust manner (Brownjohn et al. 2018; Muffat et al. 2016; Douvaras et al. 2017). To further push their transcriptional state to the primary homeostatic in vivo state, we and others have transplanted the cells into the brains of living mice, where they populated the entire brain, adopted an in vivo‐like phenotype, and could be studied for several weeks in various transgenic animals (Svoboda et al. 2019; Hasselmann et al. 2019; Xu et al. 2020; Abud et al. 2017). While this in vivo system opens many research opportunities, it bears the limitation that the human microglia are surrounded by cells of a different species' brain environment with different cell populations, cytoarchitecture, transcriptome, proteome, and metabolome than humans, potentially resulting in different physiological intercellular signalling (Miller et al. 2010; Su et al. 2004; Makalowski et al. 1996; Loomba et al. 2022). These systems also contain significant inherent complications with respect to variability and throughput. Moreover, the mice used for the transplantation were immunocompromised to allow xenograft transplantation of human cells and expressed humanized growth factors to support the maturation and survival of human microglia. This system is suboptimal for studying human microglia because the NOD‐SCID animals used carry alleles that affect the function of the innate branch of the immune system, including macrophages (Brehm et al. 2013).

To overcome the limitations inherent to chimeric systems, we devised a new platform comprised entirely of isogenic human cells in a physiological in vivo‐like brain environment, using cerebral organoid ALI‐COs, which have been shown to support more mature and diverse neural cell types and are devoid of necrotic regions that appear in cerebral organoids as they increase in size (Giandomenico et al. 2019; Qian et al. 2020). We then integrated microglia precursor cells into ALI‐COs that further differentiated and matured for up to 60 DIV and cultured in a liquid‐air interface. Microglia progenitor cells migrated into ALI‐COs within a few days, dispersed evenly throughout, and adopted a highly ramified cell morphology (Figure 1B–E; 2A,B). Homeostatic microglia are constantly surveying their microenvironment with extremely motile processes and protrusions (Nimmerjahn et al. 2005). When cultured within ALI‐COs for up to 60 days, iMG showed process motility (Movies S1, S2) and reacted to a challenge with LPS with a drastic change in their morphology from highly ramified to amoeboid‐like (Figure 1H). In combination with the morphological analysis (Figure 2A,B), transcriptional analysis (Figure 3), and this dynamic responsiveness to LPS challenge (Figure 1H), our results suggest that iMG adopt an in vivo‐like phenotype within a few days within ALI‐COs.

The choice of ALI‐COs over whole organoids was crucial for developing this model system. Whole organoids contain necrotic regions that impede the development of homeostatic microglia. It is established that brain macrophages in ischemic lesions show disintegration and amoeboid‐like morphology (Schroeter et al. 2009). Previous attempts to integrate myeloid progenitor cells into whole organoids have demonstrated lower microglia integration efficiency (Brownjohn et al. 2018; Bodnar et al. 2021; Fagerlund et al. 2021). Our observations confirmed this notion, as macrophage precursor cells adopted an amoeboid‐like morphology when added to whole organoids (Figure 1G). Yet slicing cerebral organoids and culturing them at the air‐liquid interface created an environment where induced microglia could develop an in vivo‐like phenotype.

The vast number of iMG integrated into the organoid ALI‐COs allowed us to isolate those cells in sufficient numbers for bulk RNA sequencing. The transcriptional analysis was essential to compare these cells with human primary microglia from a published transcriptomic data set of microglia that underwent RNA sequencing directly after isolation or after 1 week in vitro (Gosselin et al. 2017). We also compared iMG cultured within ALI‐COs for 10 and 60 days with iMG transplanted into the brains of living mice and that were isolated and analyzed in a very similar method and published recently by our lab (Svoboda et al. 2019). This comparison demonstrated that iMG resembled primary human microglia in the homeostatic state after 10 days or 60 days in ALI‐COs (Figure 3A). This was in contrast with iMG or primary human microglia cultured in monoculture for 7 days. Notably, the speed at which iMG acquired the in vivo‐like transcriptional profile was faster than that of iMG transplanted into mice, which required several weeks until they clustered closely with pMG. This could be due to the fact that iMG can develop better in a human brain environment as compared with the humanized mouse brain environment.

Microglia play an important role in almost all brain pathologies, yet interspecies differences and suboptimal in vitro culture conditions have imposed major limitations on modeling those diseases and testing drugs effectively. This work provides an experimental in vitro system in which PSC‐derived microglia are integrated into a 3D ALI‐CO and closely resemble the cell state of primary, homeostatic human microglia, thereby overcoming limitations of current alternative strategies to study human microglia biology. In summary, our results here allow for long‐term phenotypic analysis of human microglia as well as in‐depth genetic studies involving iPSCs from patients with various diseases or genetically modified human PSCs.

## 4 Limitations of the Study

This study focused on the morphological and transcriptional characterization of iMG in cerebral organoids and did not characterize the effects of iMG transplantation on other cell types of the cerebral organoid or the cellular composition of ALI‐COs, which has been studied before (Giandomenico et al. 2019). Notably, microglia show brain‐region‐specific differences (Grabert et al. 2016). While this study focused on microglia in cerebral organoids, other organoids such as midbrain, forebrain, or cerebellar organoids would allow the investigation of the heterogeneity of microglia and their effects on the cellular composition and cytoarchitecture in those systems (Qian et al. 2018). While we examined the effect of LPS on iMG morphology in ALI‐COs, future studies should include analyses of inflammatory mediators such as cytokines. Our morphological analysis indicated that iMG in ALI‐COs are responding to the inflammatory stimulus LPS. This approach enabled us to spatially resolve immune responses within a 3D human brain‐like environment. Although cytokine profiling was beyond the scope of this study, our findings provide a morphological framework for future work linking structural and molecular responses. While we performed extensive phenotypic characterization of key macrophage populations using IBA1 immunostaining and 3D morphology tracing, we did not conduct targeted qPCR or immunofluorescence validation of individual genes identified by RNA sequencing. Future studies are required to address this with additional experimental validation.

Moreover, we exclusively used PSCs from healthy donors, yet iPSC‐derived organoids and microglia from patients with a certain disease or gene‐edited cell lines and controls could also be employed. This would allow a broad spectrum of disease modeling and the investigation of cell‐autonomous versus nonautonomous effects of relevant mutations (Soldner and Jaenisch 2018). Another important step towards an even more complex in vitro brain environment would be the integration of a vasculature network to the cerebral organoids. Microglia have been shown to closely interact with blood vessels with relevance during development, health, and disease (Bisht et al. 2021; Hattori 2022).

## 5 Experimental Procedures

### 5.1 Generation and Culture of Human Induced Pluripotent Stem Cells

Human foreskin fibroblasts from a healthy 2‐year‐old male were ordered from the Coriell Institute (AG07095, CAT# AG07095). The reprogramming of skin fibroblasts was based on a previous method (Okita et al. 2011). Briefly, skin fibroblasts were expanded (using DMEM/F12 + 10% FBS media) and transfected by three plasmids for the expression of Yamanaka reprogramming factors (pCXLE‐hSK, Addgene #27078; pCXLE‐hOCT3/4‐shp53‐F, Addgene #27077; and pCXLE‐hUL, Addgene #27080) using Bio‐Rad GenePulser X‐Cell. After 7 days, transfected cells were transferred onto irradiated mouse embryonic fibroblasts (MEF) and further cultured in ReproTeSR media (StemCell Technologies). After 25–30 days of transfection, iPSC clones were manually picked, expanded on Matrigel‐coated plates with mTeSR1 media, and characterized. After five passages, the iPSC line was subjected to karyotyping to exclude chromosomal abnormalities. After 15–20 passages, the experiments were conducted. As a control, the already established and characterized ES cell line H1 was used (RRID:CVCL_9771, Lab of William Murphy at University of Wisconsin‐Madison, http://www.ebi.ac.uk/efo/EFO_0003042).

Once established, hiPSCs were cultured at 37C in 5% CO_2_ on Matrigel‐coated plates in Gibco *StemFlex Medium and passaged with ReLeSR* (Stemcell technologies) every 5 days. Cells were frozen in media containing 10% DMSO. Cells were tested negative for mycoplasma twice per year and checked for bacterial or fungal contamination during every media change. EGFP was targeted into the *AAVS1* locus via nucleofection combining an EGFP targeting plasmid (Addgene; catalog no. 22212) and a human codon‐optimized SpCas9 and chimeric guide RNA expression plasmid (Addgene; catalog no. 42230), into which the Target Guide Sequence was cloned into using the oligos: Oligo 1: CACCGGGGGCCACTAGGGACAGGAT, Oligo 2: AAACATCCTGTCCCTAGTGGCCCCC. After puromycin selection, EGFP‐expressing clones were manually picked. Clones homozygous for the insertion in the *AAVS1* locus were selected for further experimentation. All experiments involving cells from human subjects were performed in compliance with MIT COHUES protocol 0612002068R012 (Wogram et al. 2024).

### 5.2 Southern Blot

Genomic DNA (15 μg) was digested with SphI, separated on a 0.8% agarose gel, transferred to a nylon membrane (Amersham), and hybridized with ^32^P internal and external probes labeled by Prime‐It II Random Primer Labeling Kit (Agilent Technologies, Cat#300385) (Wogram et al. 2024).

### 5.3 Myeloid Precursor Cell Differentiation

Using an established protocol, PSCs were differentiated into microglia precursors (Brownjohn et al. 2018). In brief, PSCs were dissociated with TrypLE Express (Gibco) and allowed to form EBs for 4 days containing 10,000 cells each in Stemflex media supplemented with 10 μM ROCK inhibitor, 50 ng/mL BMP4 (Peprotech), 20 ng/mL SCF (Peprotech), and 50 ng/mL VEGF‐165 (Peprotech) with half a media change after 2 days without ROCK inhibitor. Subsequently, EBs were cultured in X‐VIVO (Fisher Scientific, #BW04418Q), supplemented with 2 mM GlutaMax, 100 U/mL penicillin, and 100 mg/mL streptomycin (VWR, #12001‐692), 55 μM b‐mercaptoethanol (Sigma Aldrich), 100 ng/mL M‐CSF (Peprotech), and 25 ng/mL IL‐3 (Peprotech). From this point on, the medium was exchanged every 4–7 days, and cells in suspension were harvested and used as microglia precursor cells (iMPCs), which were either transplanted or further cultured in microglia differentiation media containing Neurobasal (Life Technologies, #21103049) supplemented with Gem21 without Vitamin A (Gemini Bio‐Products, #400161), 2 mM GlutaMax (Life Technologies), 100 U/mL penicillin, 100 μg/mL streptomycin, 100 ng/mL IL‐34 (Peprotech), and 10 ng/mL M‐CSF (Peprotech) with media changes every 5–7 days.

### 5.4 ALI‐CO Generation and Microglia Transplantation

Cerebral organoids were differentiated from human PSCs according to the manufacturer's instructions with the STEMdiff Cerebral Organoid Kit (Catalog #08570). After 38–45 days in maturation media in a six‐well plate on an orbital shaker rotating at 95 rpm at 37C, the media was replaced by neurobasal media (Life Technologies, #21103049) supplemented with Gem21 without Vitamin A (Gemini Bio‐Products, #400161), 2 mM GlutaMax (Life Technologies), 100 U/mL penicillin, and 100 μg/mL streptomycin with complete media changes every 3–5 days. Once the organoid sections reached a diameter of 2 mm, the organoids were sliced with a McIllwain tissue chopper in 350 μm sections. After slicing, sections were washed in an ice‐cold cutting solution consisting of HBSS containing 0.6% glucose, 15 mM HEPES buffer, 100 U/mL penicillin, and 100 μg/mL streptomycin. Intact sections were further cultured and sliced every month. After three rounds of sectioning, a maximum of five sections were placed on cell culture inserts (Fisher Scientific, PICM0RG50) in six‐well plates with complete media changes every 2–3 days, similar to published protocols published by (Giandomenico et al. 2019; Qian et al. 2020).

ALI‐COs were cultured for 7 days on the inserts to recover before a drop of 2 μL containing 4000 MPCs was carefully placed on top of a section using a 10‐μL pipette tip. For transplantation, MPCs were used up to 40 days after differentiation initiation.

iMPCs were added to uncut organoids by culturing one uncut organoid in an ultra‐low attachment 96‐well plate well (Corning) for 24 h together with 120,000 MPCs in 200 μL media.

### 5.5 LPS Treatment

For LPS stimulation, 100 ng/mL LPS (Sigma Aldrich, CAS#L2630‐25MG) was added to the media for 48 h before analysis.

### 5.6 Live Cell Imaging

Low magnification live cell imaging was performed on ALI‐COs with a Zeiss 710 confocal microscope at 37C, 5% CO_2_. High magnification live cell imaging was performed on ALI‐COs with a Nikon Ti automated inverted microscope with incubation enclosure at 37C, 5% CO_2_.

### 5.7 Immunohistochemistry

Tissues were fixed in 10% formalin, washed in PBS, and blocked in PBS containing 5% donkey serum and 0.3% Triton X‐100. Primary antibodies were the mouse monoclonal antibody against Iba1 (Abcam, Cat# ab283319, RRID:AB_2924797), the chicken polyclonal antibody against GFP (Aves Labs Cat# GFP‐1020, RRID:AB_10000240), the chicken polyclonal antibody against MAP 2A/B (EnCor Biotechnology Cat# CPCA‐MAP 2, RRID:AB_2138173), and the mouse monoclonal antibody against Tubulin beta 3 (BioLegend Cat# 801201, RRID:AB_2313773). Primary antibodies were added for 72 h at 4C. After washing in PBS, secondary antibodies were added in PBS for 48 h at 4C. After washes, DAPI was added to stain nuclei for 1 h at room temperature. Sections were mounted using fluoromount‐G (ThermoScientific).

### 5.8 Image Acquisition

Images were acquired on a Zeiss 710 confocal microscope. For full‐section images, tile scans were performed of three different sections with a 10× objective, combined with a *Z*‐stack through the entire section with a 9‐μm interval. For images used for morphological measurements, tile scans of at least four fields of view were taken using the 40× objective, with a *Z*‐stack through the entire section at an interval of 500 nm.

### 5.9 Image Visualization

Images were cropped, merged and optimized with ImageJ (RRID:SCR_003070), and arranged with Adobe Illustrator 2022 (RRID:SCR_010279).

### 5.10 iMG Isolation and FACS

EGFP^+^ microglia cells were isolated from organoid sections according to an established protocol in the lab (Svoboda et al. 2019). Briefly, organoid sections were washed twice in 4C PBS and homogenized in a 2‐mL douncer in ice‐cold HBSS (GIBCO), supplemented with glucose (0.5%) and RNAsin (1/1000; Promega; N2615), and passed through a 70‐μm cell strainer (FALCON). Four to six organoid sections were homogenized together and treated as one replicate to obtain an iMG number high enough for downstream analysis. Dissociated cells were centrifuged 400 g for 10 min at 4C, and pellets were resuspended in a 500‐μL FACS buffer (PBS supplemented with 1% bovine serum albumin (BSA) (Sigma‐Aldrich), EDTA (2 mM), and HEPES (25 mM; ThermoScientific). A FACS buffer also contained DAPI (Life Technologies) to indicate dead cells.

Using a BD Aria I sorter, gates were set to isolate live singlets using FSC/SSC/DAPI fluorescent properties. GFP gating was established using a negative control cerebral organoid or neural culture containing no microglia for every experiment. For bulk RNAseq experiments, GFP+ cells were sorted directly into an RLT+ lysis buffer (Qiagen) containing 1% β‐mercaptoethanol. Total RNA was isolated using the Qiagen RNeasy‐micro+ kit and stored in RNAse‐free water at −80C until further processing.

### 5.11 RNAseq

There were six sample groups (iMPCs, 10d iMG, 60 DIV iMG, ALI‐CO‐iMG 10d, ALI‐CO‐iMG 60d, ALI‐CO‐iMG 60d H1) with *n* = 6 for each sample set, except for ALI‐CO‐iMG 60d H1 for which four replicates were used. ALI‐CO‐iMG 10d and 60d samples originated from different collection of iMPCs, as did the iMPC group. MPCs were collected from the suspension, sorted, and used for RNAseq, see section “Myeloid Precursor Cell Differentiation” or Brownjohn et al. (2018). One n corresponds to all collected cells pooled from 4 to 6 organoid sections for cells isolated from organoid sections. For 10 and 60d iMG samples, one n corresponds to two pooled wells of a six‐well plate, with one plate generating three samples.

RNA was collected using the Qiagen MicroRNeasy+ kit according to the manufacturer's protocol. For library preparation, 3 ng of RNA was used as input using Takara's SMART‐Seq v4 Ultra Low Input RNA protocol per manufacturer's guidelines. Briefly, RNA underwent reverse transcription via template switching and amplification, resulting in double‐stranded cDNA. The amount of cDNA between 100 and 3000 bp was calculated by Fragment Analyzer and Qubit. 150 pg of the sample in range was added to Illumina's Nextera XT and processed according to the manufacturer's guidelines. Briefly, the cDNA underwent tagmentation using Nextera XT's transposase, and unique dual indexes were added by PCR amplification. Final libraries went through QC with the Fragment Analyzer and qPCR on a Roche Light Cycler 480 II. All libraries were then pooled and sequenced at single‐end 40 base‐pair using the NOVASEQS4 and the SMARTer v.4 protocol.

### 5.12 Quantification and Statistical Analysis

#### 5.12.1 Immunohistochemical Quantification

A minimum of three images were taken per ALI‐CO, and the images were quantified and averaged together, using a minimum of 45 cells from at least three ALI‐COs. The resulting average values were used in the final statistical analysis with each n representing one ALI‐CO. Automated filament tracing was performed using Imaris version 2 in the GFP channel. For filament tracing, Imaris version 2 was set to detect spherical starting points with a size of 5 μm and seed point size of 1 μm for all ALI‐COs. Default settings were used as a threshold for GFP intensity to calculate dendrite volume. Significance was determined using the two‐tailed unpaired *t* test with Graphpad Prism 10.5.0.

#### 5.12.2 RNAseq Analysis

Within 2 months after the RNA sequencing, reads were aligned to the human (GRCh38) genomes using STAR v2.7.1a (indexed using Ensembl 96 transcript models). Gene levels were determined with the methods used by Svoboda et al. (2019). Briefly, SAM files were converted to HOMER tags, which were used (with Ensembl 96 transcript models) to generate raw counts with HOMER's analyzeRepeats.pl. Genes profiled were compared with those from Gosselin et al. (2017) and Svoboda et al. (2019). Raw counts (Table S2) and genes present in both experiments were used for further analysis. Raw counts from both datasets were normalized with DESeq2 v1.22.2 using fitType = “mean,” log2‐transformed, and then batch‐corrected with ComBat (8) (with no covariates) using the sva v3.34.0 package in R. Before performing PCA, counts underwent variance stabilizing transformation. Using batch‐corrected continuous values, differential expression was assayed using limma v3.42.2 with default settings. Before correlation analysis, replicate normalized gene profiles were median‐summarized, and one pseudocount was added before log2‐transformation. Gene Ontology analysis was done by loading a list of the genes increased or decreased by twofold or more between 60 dpi in vivo iMG and 60d in vitro iMG into the g:GOSt tool in g:Profiler (Sep 2022 version). For the comparison with Svoboda et al. (2019), gene counts were quantile‐normalized, compared as log2 ratios, and matched with our sample genes by gene symbol. For scatterplots, subsets of genes were obtained as before, analyzing only differentially expressed or microglial‐specific genes.

Sample distances were calculated in two‐dimensional (Euclidean) principal component space (PC1 and PC2) using all vs. all distances between replicates, with differences assayed by ANOVA, followed by pairwise comparisons with Tukey's HSD (reporting adjusted *p*‐values).

## Author Contributions

E.W., F.S., A.K., A.F., D. F, G.W.B, and R.J. were responsible for conceptualization, writing, and review. E.W., F.S., D.F., G.W.B., A.K., and A.F. were responsible for investigation, data curation and analysis, and methodology. E.W., F.S., D.F., G.W.B. A. K, and A.F. performed the experiments and assisted with data analysis.

## Conflicts of Interest

R.J. is an advisor/co‐founder of Fate Therapeutics and Fulcrum Therapeutics. A.F. is a co‐founder and shareholder of StemAxon.

## Supporting information




**Dataset: S1** Tables containing differentially expressed genes between 60d versus 60 DIV, 60d versus 10d.

**Figure S1:** Cerebral organoid ALI‐CO contained neurons, synaptic elements, neural precursor cells, and oligodendrocytes. (A) Confocal microscopy after immunohistochemistry against GFP (green), the neurofilament marker SMI312 (red), and DAPI (blue) in an ALI‐CO containing ALI‐CO‐iMG 10d. (B) Confocal microscopy after immunohistochemistry against GFP (green), the presynaptic marker Synapsin I (red), and DAPI (blue) in an ALI‐CO containing ALI‐CO‐iMG 10d. (C) Confocal microscopy after immunohistochemistry against GFP (green), the neural precursor and astroglial marker Nestin (red), and DAPI (blue) in an ALI‐CO containing ALI‐CO‐iMG 10d. (D) Confocal microscopy after immunohistochemistry against the oligodendroglial markers MBP (green) and Olig2 (red), and DAPI (blue) in an ALI‐CO containing no iMG. Scale bars: 100 μm.

**Movie S1:** Low magnification live cell imaging of process motility in ALI‐CO‐iMG 10d (acquisition: frame interval 37 s; display: 7.5 frames per second). Scale bar: 100 μm.

**Movie S2:** High magnification live cell imaging of process motility in a ALI‐CO‐iMG 60d (acquisition: frame interval 15 s; display: 2.5 frames per second). Scale bar: 50 μm.

**Data S1:** Supporting Information.

## Data Availability

All data needed to evaluate the conclusions in the paper are present in the paper and/or the Supporting Information. Information and requests for biological resources, reagents, and data should be directed to and will be fulfilled by the lead contacts, R.J. (jaenisch@wi.mit.edu). All unique materials generated in this study are available from the lead contacts by reasonable request, but we may require a completed materials transfer agreement. The FIAR and CARE data management principles were followed.
