# Supplementary material for: Human iPSC‐Derived Microglia Integrate Into Cerebral Organoids and Assume an In Vivo‐Like Phenotype
Source: Eur J Neurosci. 2025 Nov 12;62(9):e70281. doi: 10.1111/ejn.70281 (PMC12606696; doi:10.1111/ejn.70281)
Supplement: Supplementary file 2 — Figure S1: Cerebral organoid ALI‐CO contained neurons, synaptic elements, neural precursor cells, and oligodendrocytes. (A) Confocal microscopy after immunohistochemistry against GFP (green), the neurofilament marker SMI312 (red), and DAPI (blue) in an ALI‐CO containing ALI‐CO‐iMG 10d. (B) Confocal microscopy after immunohistochemistry against GFP (green), the presynaptic marker Synapsin I (red), and DAPI (blue) in an ALI‐CO containing ALI‐CO‐iMG 10d. (C) Confocal microscopy after immunohistochemistry against GFP (green), the neural precursor and astroglial marker Nestin (red), and DAPI (blue) in an ALI‐CO containing ALI‐CO‐iMG 10d. (D) Confocal microscopy after immunohistochemistry against the oligodendroglial markers MBP (green) and Olig2 (red), and DAPI (blue) in an ALI‐CO containing no iMG. Scale bars: 100 μm. [file EJN-62-0-s004.pdf]

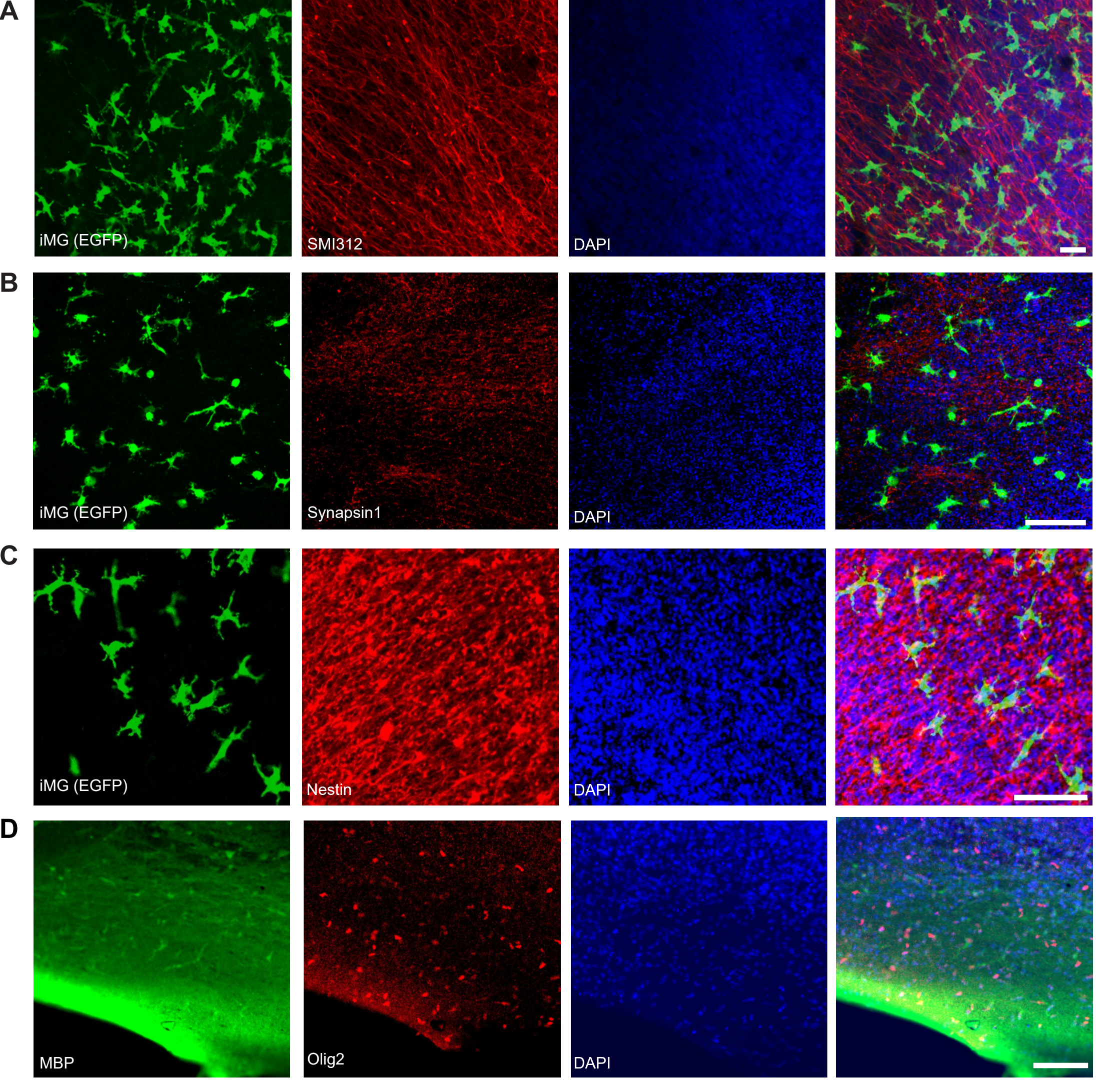

Supplementary Fig. 1: Cerebral organoid ALI-COs contained neurons, synaptic elements, neural precursor cells, and oligodendrocytes.

(A) Confocal microscopy after immunohistochemistry against GFP (green), the neurofilament marker SMI312 (red), and DAPI (blue) in an ALI-CO containing ALI-CO-iMG 10d.

(B) Confocal microscopy after immunohistochemistry against GFP (green), the presynaptic marker Synapsin I (red), and DAPI (blue) in an ALI-CO containing ALI-CO-iMG 10d.

(C) Confocal microscopy after immunohistochemistry against GFP (green), the neural precursor and astroglial marker Nestin (red), and DAPI (blue) in an ALI-CO containing ALI-CO-iMG 10d.

(D) Confocal microscopy after immunohistochemistry against the oligodendroglial markers MBP (green) and Olig2 (red), and DAPI (blue) in an ALI-CO containing no iMG. Scale bars: 100 µm.
