# Supplementary material for: Human iPSC‐Derived Microglia Integrate Into Cerebral Organoids and Assume an In Vivo‐Like Phenotype
Source: Eur J Neurosci. 2025 Nov 12;62(9):e70281. doi: 10.1111/ejn.70281 (PMC12606696; doi:10.1111/ejn.70281)
Supplement: Supplementary file 5 — Data S1: Supporting Information. [file EJN-62-0-s002.pdf]

KEY RESOURCES TABLE

REAGENT or RESOURCE	SOURCE	IDENTIFIER
Antibodies		
Chicken polyclonal antibody against GFP	Aves Labs	Cat# GFP-1020
Mouse monoclonal antibody against Iba1	Abcam	Cat# ab28331; RRID:AB_2924797
Chicken polyclonal antibody against MAP2A/B	EnCor Biotechnology	Cat# CPCA-MAP2; RRID:AB_2138173
Mouse monoclonal antibody against TUJ1/TUBB3	Biolegend	Cat# 801201; RRID:AB_2313773
Chemicals, Peptides, and Recombinant Proteins		
y-27632	Thermo Fisher Scientific	CAT#688000100MG
Recombinant human BMP4	Peprotech	CAT#120-05ET
Recombinant human SCF	Peprotech	CAT#300-07
Recombinant human VEFG-165	Peprotech	CAT#100-20-50UG
Recombinant human M-CSF	Peprotech	CAT#AF-300-25
Recombinant human IL-3	Peprotech	CAT#200-03
Recombinant human IL-34	Peprotech	CAT#200-34-500UG
Levetiracetam	Sigma Aldrich	CAT#L8668-50MG
Gem21 without Vitamin A	Gemini Bio-Products	CAT#400161
X-VIVO media	Fisher Scientific	CAT#BW04418Q
Advanced DMEM/F-12	Life Technologies	CAT#12634010
FBS	Hyclone	CAT#SH30396.03
SphI-HF	New England Biolabs	CAT#R3182M
Hybond-xl Membrane	Fisher Scientific	CAT#45001151
Prime-It II Random Primer Labeling Kit	Agilent Technologies	CAT#300385
TrypLE Express	Life Technologies	CAT#12605010
StemFlex Medium	Life Technologies	CAT#A3349401
mTeSR 1 Medium	STEMCELL Technologies	CAT#5850
Matrigel Matrix	Fisher Scientific	CAT#CB40234
Glutamax	Life Technologies	CAT#3505006
DAPI (4',6-Diamidino-2-Phenylindole, Dihydrochloride)	Life Technologies	CAT#D1306
RNAsin	Promega	CAT#N2615
HBSS	Life Technologies	CAT#14175095
Glucose solution 45% in H2O	Sigma Aldrich	CAT#G8769-100ML
Bovine Serum Albumin fatty acid free	Sigma Aldrich	CAT#A8806-5G
UltraPure 0.5 M EDTA	Life Technologies	CAT#15575020
HEPES solution	Sigma Aldrich	CAT#H0887-20ML
RNeasy Mini Kit	Qiagen	CAT#74034
2-Mercaptoethanol	Sigma Aldrich	CAT#M3148-25ML
Nuclease-free Water	Life Technologies	CAT#AM9937
Lipopolysaccharides	Sigma Aldrich	CAT#L2630-25MG
Neurobasal media	Life Technologies	CAT#21103049

Penicillin-Streptomycin	VWR	CAT#45000-652
Critical Commercial Assays		
STEMdiff Cerebral Organoid Kit	STEMCELL Technologies	CAT#08570
SMART-Seq v4 Ultra Low Input RNA Kit for Sequencing	Takarabio	CAT#634888
Deposited Data		
Human iPSC-derived microglia in mouse brain bulk RNAseq	Svoboda et al. 2019	GEO:GSE139192
Human primary microglia bulk RNAseq	Gosselin et al. 2017	GEO:GSE89960
Human iPSC-derived microglia in cerebral organoid slice cultures	This study	GEO:GSE222754
Experimental Models: Cell Lines		
Human foreskin fibroblasts	Coriell Institute	AG07095
Human embryonic stem cell line H1	Lab of William Murphy at University of Wisconsin-Madison	RRID:CVCL_9771
Oligonucleotides		
Target Guide Sequence Oligo 1: CACCGGGGGCCACTAGGGACAGGAT	Sigma	N/A
Target Guide Sequence Oligo 2: AAACATCCTGTCCCTAGTGGCCCCC	Sigma	N/A
Recombinant DNA		
pCXLE-hSK	Addgene	pCXLE-hSK was a gift from Shinya Yamanaka (Addgene plasmid # 27078 ; http://n2t.net/addgene:27078 ; RRID:Addgene_27078)
pCXLE-hOCT3/4-shp53-F	Addgene	pCXLE-hOCT3/4-shp53-F was a gift from Shinya Yamanaka (Addgene plasmid # 27077 ; http://n2t.net/addgene:27077 ; RRID:Addgene_27077)
pCXLE-hUL	Addgene	pCXLE-hUL was a gift from Shinya Yamanaka (Addgene plasmid # 27080 ; http://n2t.net/addgene:27080 ; RRID:Addgene_27080)
AAV-CAGGS-EGFP	Addgene	AAV-CAGGS-EGFP was a gift from Rudolf Jaenisch (Addgene plasmid # 22212 ; http://n2t.net/addgene:222

		12 ; RRID:Addgene_22212)
pX330-U6-Chimeric_BB-CBh-hSpCas9	Addgene	pX330-U6-Chimeric_BB-CBh-hSpCas9 was a gift from Feng Zhang (Addgene plasmid # 42230 ; http://n2t.net/addgene:42230 ; RRID:Addgene_42230)
Software and Algorithms		
ImageJ (Fiji)	https://imagej.net/software/fiji/downloads	RRID:SCR_003070
Imaris	Bitplane	RRID:SCR_007370
limma v3.42.2	Gordon Smyth	RRID:SCR_010943
DESeq2 v1.22.2	Michael Love	RRID:SCR_015687
ZEN Microscopy Software	Zeiss	RRID:SCR_013672
MetaMorph Microscopy Automation and Image Analysis Software	Moleculardevices	RRID:SCR_002368
G:Profiler	Uku Raudvere	RRID:SCR_006809
STAR v2.7.1a	A. Dobin	RRID:SCR_004463
Ensemble	http://www.ensembl.org/index.html	RRID:SCR_002344
sva packadge	Leek JT	RRID:SCR_012836
Other		
Corning Costar Ultra-Low Attachment Multiple Well Plate size 96 well, clear round bottom	Sigma Aldrich	CAT#CLS7007-24EA
Transfer pipets	Weber Scientific	CAT#3017-18
70 um cell strainer	VWR	CAT#21008-952
DOUBLE EDGE BLADES	VWR	CAT#100491-886
Millicell™ Culture Plate Inserts	Thermo Fisher Scientific	CAT#PICM0RG50
